# Dual Profile of Environmental Enrichment and Autistic-Like Behaviors in the Maternal Separated Model in Rats

**DOI:** 10.3390/ijms22031173

**Published:** 2021-01-25

**Authors:** Monireh Mansouri, Hamidreza Pouretemad, Gregers Wegener, Mehrdad Roghani, Masoud Afshari, Carina Mallard, Maryam Ardalan

**Affiliations:** 1 Department of Cognitive Psychology, Institute for Cognitive and Brain Sciences, Shahid Beheshti University, Tehran 1983969411, Iran; monirehmansouri@clin.au.dk (M.M.); H-Pouretemad@sbu.ac.ir (H.P.); ma_afshari@sbu.ac.ir (M.A.); 2 Translational Neuropsychiatry Unit, Department of Clinical Medicine, Aarhus University, 8000 Aarhus, Denmark; wegener@clin.au.dk; 3 Center of Excellence in Cognitive Neuropsychology, Institute for Cognitive and Brain Sciences, Shahid Beheshti University, Tehran 1983969411, Iran; 4 Department of Psychology, Shahid Beheshti University, Tehran 1983969411, Iran; 5 Center of Excellence for Pharmaceutical Sciences, North-West University, Potchefstroom 2520, South Africa; 6 AUGUST Centre, Department of Clinical Medicine, Aarhus University, 8240 Risskov, Denmark; 7 Neurophysiology Research Center, Shahed University, Tehran 3319118651, Iran; mehjour@yahoo.com; 8 Centre for Perinatal Medicine and Health, Institute of Neuroscience and Physiology, Sahlgrenska Academy, University of Gothenburg, 50530 Gothenburg, Sweden; carina.mallard@neuro.gu.se

**Keywords:** animal model of autism, neonatal isolation, environmental enrichment, brain plasticity, BDNF

## Abstract

**Background**: Environmental Enrichment (EE) has been suggested as a possible therapeutic intervention for neurodevelopmental disorders such as autism. Although the benefits of this therapeutic method have been reported in some animal models and human studies, the unknown pathophysiology of autism as well as number of conflicting results, urge for further examination of the therapeutic potential of EE in autism. Therefore, the aim of this study was to examine the effects of environmental enrichment on autism-related behaviors which were induced in the maternal separation (MS) animal model. **Material and Methods**: Maternally separated (post-natal day (PND) 1–14, 3 h/day) and control male rats were at weaning (PND21) age equally divided into rats housed in enriched environment and normal environment. At adolescence (PND42–50), the four groups were behaviorally tested for direct social interaction, sociability, repetitive behaviors, anxiety behavior, and locomotion. Following completion of the behavioral tests, the blood and brain tissue samples were harvested in order to assess plasma level of brain derived neurotrophic factor (BDNF) and structural plasticity of brain using ELISA and stereological methods respectively. **Results**: We found that environmental enrichment reduced repetitive behaviors but failed to improve the impaired sociability and anxiety behaviors which were induced by maternal separation. Indeed, EE exacerbated anxiety and social behaviors deficits in association with increased plasma BDNF level, larger volume of the hippocampus and infra-limbic region and higher number of neurons in the infra-limbic area (*p* < 0.05). **Conclusion**: We conclude that environmental enrichment has a significant improvement effect on the repetitive behavior as one of the core autistic-like behaviors induced by maternal separation but has negative effect on the anxiety and social behaviors which might have been modulated by BDNF.

## 1. Introduction

The effect of environmental factors on brain development has long been known [1]. Poverty or enrichment of the environment, stress, nutrition and many other factors affect structural, cellular and network connections in the brain, and thereby contribute to individuals’ future cognitive behaviors and potential psychological disorders [2,3,4,5]. Autism Spectrum Disorder (ASD) is a neurodevelopmental disorder with a global prevalence of more than 1% and is characterized by deficits in social and stereotyped behaviors. Despite its prevalence, no definitive treatment has been identified [6]. Autism is commonly recognized as a highly genetic disorder [7,8], but recently, it was found that environmental factors such as early life maternal separation (MS) have significant impact on the risk of autism [9,10,11,12]. However, it is not clear to what extent the environmental factors influence the expression of autistic symptoms. Some researchers proposed that one of the reasons for the increase in the prevalence of autism is a change in the lifestyle of families and the high use of digital devices among children from an early age as one of the factors resulting inadequate parent child interaction [10,13]. They suggested enrichment of the environment as a potential compensatory factor for the negative effects of such an adverse environmental complication. This non-pharmacological intervention has been used in animal models of neurodevelopmental and neurodegenerative disorders [14], mainly as models of behavioral intervention [15]. The findings pointed to the possibility that adding different objects to the animal’s environment through increasing the level of complexity and novelty, enhancement of sensory stimulation, and stimulating cognitive activity and physical training may modify pathophysiological mechanisms, and manifests as an improvement of autistic symptoms [16]. Indeed, preclinical studies showed that rearing in an enriched environment suppressed the expression of some autistic behavioral phenotypes [17,18,19,20]. Unfortunately, not all studies are in line with these suggestions, as were observed by lack of recovery and exacerbations of social behavior deficits and anxiety following environmental enrichment (EE) in different studies [21,22,23].

In our recent study, we observed the beneficial effect of oxytocin on the improvement of autistic-like behaviors with an effect on brain plasticity along with a decrease in plasma BDNF (brain derived neurotrophic factor) levels in a maternal separate rat model of autism [24]. We showed that changes in the plasma BDNF level could be associated with changes in the brain plasticity and improvement in autistic-like symptoms. Accordingly, we decided to investigate the effect of EE on the mentioned parameters in the current experiment. Therefore, the main purpose of this study was to investigate the effect of environmental enrichment on (i) autistic-like behaviors which were induced by maternal separation, (ii) brain plasticity, and (iii) the plasma BDNF level which is also altered in the ASD patients [25] and also as the main neurotrophic factor involved in brain plasticity.

## 2. Results

### 2.1. Repetitive Behaviors

Repetitive behaviors were assessed using self-grooming and marble burying tests.

#### 2.1.1. Self-Grooming 

**Figure 1:**
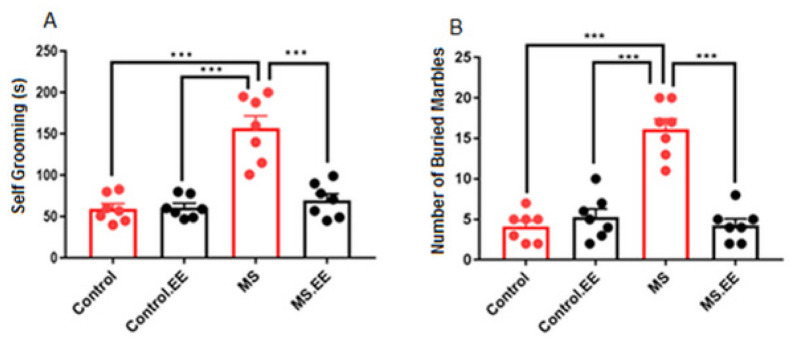
Effects of maternal separation and EE on the stereotypic behaviors. (**A**): self-grooming, (**B**): marble burying. *** *p* < 0.001. *N* = 10 in each group. Data are presented before log-transformation, MS: Maternal Separation, EE: Environmental Enrichment.

In the open field test, the time spent for self-grooming was recorded as a repetitive behavior. Results of two-way ANOVA analysis showed that there was a significant interaction between MS and EE on the time spent for self-grooming (*F*_(1.24)_ = 18.32, *p* < 0.001) with significant main effects of MS and EE (*F*_(1.24)_ = 29.28, *p* < 0.001 and *F*_(1.24)_ = 14.91, *p* = 0.001), respectively. Moreover, the maternal separation group spent significantly more time for self-grooming than the other three groups (*p* < 0.001). There was no significant difference between the other groups (*p* > 0.05) (Figure 1A).

#### 2.1.2. Marble Burying

There was a significant interaction between the effects of MS and EE (*F*_(1.24)_ = 44.76, *p* < 0.001) on the burying of marbles. Simple main effects of MS and EE were significant (*F*_(1.24)_ = 32.04, *p* < 0.001 and *F*_(1.24)_ = 30.40, *p* < 0.001), respectively. Maternal separated rats significantly buried a greater number of marbles than the other three groups (Figure 1B, *p* < 0.001). Indeed, the above results showed an increased repetitive behavior in maternal separated rats which were significantly improved by environmental enrichment. 

### 2.2. Social Behavior

In the three-chamber test, two social behaviors including sociability and direct social interaction were examined. 

#### 2.2.1. Sociability

**Figure 2:**
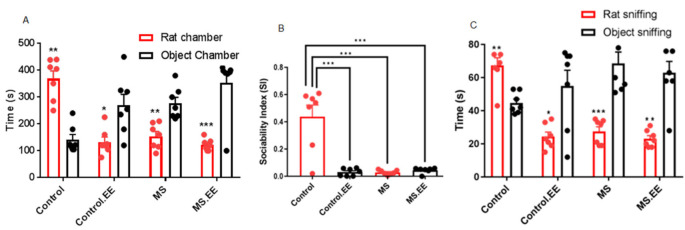
Effects of maternal separation and EE on social behavior (**A**), sociability index (**B**) and direct social interaction (**C**). * *p* < 0.05, ** *p* < 0.01, *** *p* < 0.001. *N* = 10 in each group. MS: Maternal Separation, EE: Environmental Enrichment.

Comparison of the time spent in stranger rat chamber with non-social object chamber in this test was used as a sociability index (SI). Within group comparisons showed that in the control group, the time spent in the stranger rat chamber was significantly longer than in the non-social object chamber (*p* = 0.003). While in the MS group, the time spent in the stranger rat chamber was significantly less than in the non-social object chamber (*p* = 0.005). There was no significant improvement in the social behavior in the MS.EE group as the time spent in the non-social object chamber was significantly longer than in the stranger rat chamber *(p* = 0.001). Interestingly, in the control group with enriched environment, sociability was significantly reduced, and the time spent in the non-social object chamber was significantly longer than in the stranger rat chamber (*p* = 0.035) (Figure 2A).

Social index calculation (time exploring social chamber−time exploring non-social chamber)/(time exploring social chamber + time exploring non-social chamber) confirmed the above result. Two way ANOVA analysis showed that MS and EE had significant main effects on the social index (*F*_(1,24)_ = 26.53, *p <* 0.001 and *F*_(1,24)_ = 28.57, *p* < 0.001). Tukey post hoc test showed significant decrease in the SI index in the control.EE, MS, and MS.EE groups in comparison with control group (*p* < 0.001) (Figure 2B). 

#### 2.2.2. Direct Social Interaction

Durations of direct sniffing of testing rat with stranger rat and with empty cage were recorded as a direct social interaction behavior. The results showed that in the control group, time spent sniffing stranger rat was significantly higher (*p* = 0.002), but in the control.EE, MS, and MS.EE groups, the time spent sniffing stranger rat was significantly lower than sniffing the empty cage (*p* = 0.024, *p* = 0.001, *p* = 0.002, respectively) (Figure 2C).

#### 2.2.3. Anxiety-Like Behavior

**Figure 3:**
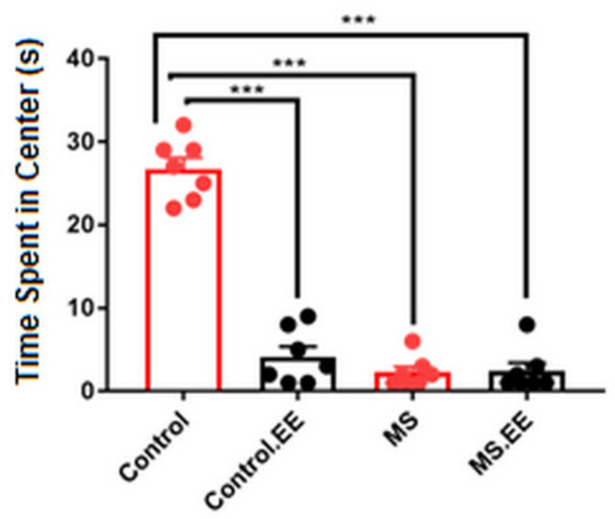
Effects of maternal separation and EE on anxiety behavior in the open field test. *** *p* < 0.001. *N* = 10 in each group. MS: Maternal Separation, EE: Environmental Enrichment.

The time spent in the central circle of the open-field test box was recorded as a measure of anxiety behavior in rats. Less time spent in the central circle was indicative of an anxiety behavior in rats. Here, we observed a significant interaction between MS and EE (*F*_(1.24)_ = 48.24, *p* < 0.001) on the anxiety behavior and significant simple main effects of MS and EE (*F*_(1.24)_ = 64.92, *p* < 0.001; *F*_(1.24)_ = 46.97, *p* < 0.001) on the anxiety behavior. All groups of MS, MS.EE, and control.EE were present in the central circle for significantly less time than the control group (*p* < 0.001) (Figure 3).

### 2.3. Motor Behavior

In the open field test, number of times the lines were crossed by rat were recorded as a locomotion behavior. Data analysis by using two-way ANOVA showed no significant interaction between MS and EE (*F*_(1.24)_ = 0.92, *p* = 0.345). Accordingly, motor behavior did not significantly change following MS and/or EE (data not shown). 

#### Plasma BDNF Level

**Figure 4:**
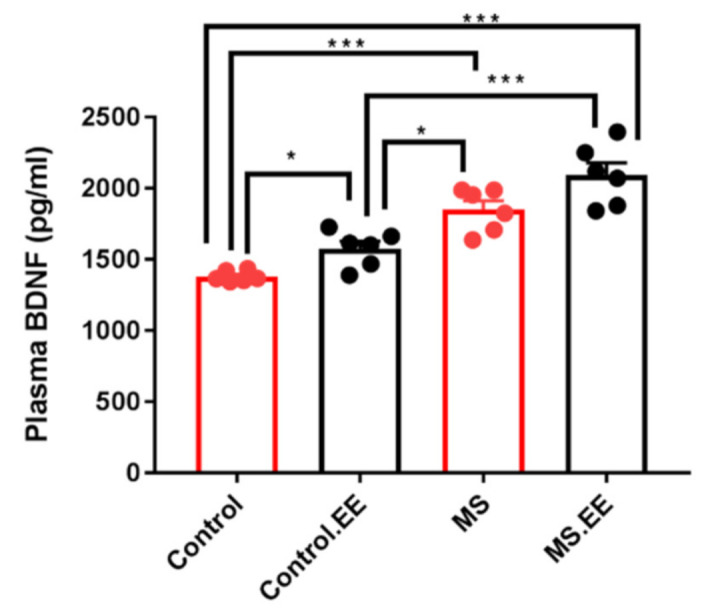
Effects of maternal separation and EE on plasma brain derived neurotrophic factor (BDNF) levels. * *p* < 0.05, *** *p* < 0.001. *n* = 6 in each group. Data are presented before log-transformation, MS: Maternal Separation, EE: Environmental Enrichment.

The results revealed no significant interaction of MS and EE (*F*_(1.20)_ = 0.02, *p* = 0.88) but significant main effects of MS (*F*_(1.20)_ = 79.79, *p* < 0.001) and EE (*F*_(1.20)_ = 15.60, *p* = 0.001) on the BDNF plasma level. Tukey post hoc test showed that the plasma level of BDNF was significantly increased in MS, MS.EE, and control.EE groups compared to the control group (*p* < 0.001, *p* < 0.001, *p* = 0.041). MS and MS.EE groups showed also significant differences from control.EE group (*p* = 0.05, *p* < 0.001) but there was no significant difference between MS and MS.EE groups (*p* > 0.05) (Figure 4). 

### 2.4. Quantitative Stereological Measurements

#### 2.4.1. Effects of MS and EE on the Volume of the Hippocampus and Its Sub-Regions

**Figure 5:**
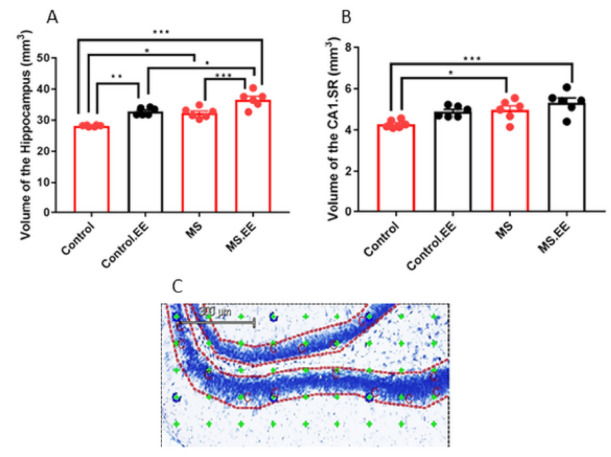
Effects of maternal separation and EE on the hippocampus (**A**) and CA1.SR volumes (**B**) * *p* < 0.05, ** *p* < 0.01, *** *p* < 0.001. Illustration of Cavalieri estimator using point counting to measure the volume of the GCL sub-region of hippocampus (objective lens 10×) (**C**). *N* = 6 in each group. MS: Maternal Separation, EE: Environmental Enrichment.

The results showed significant main effects of MS (*F*_(1.20)_ = 24.12, *p* < 0.001) and EE (*F*_(1.20)_ = 38.22, *p* < 0.001) on the total volume of the hippocampus. Regarding subregions of hippocampus, we observed no effects of MS and EE on the volume of GCL and MDG (*F*_(1.20)_ = 3.01, *p* = 0.09), (*F*_(1.20)_ = 0.456, *p* = 0.50), (*F*_(1.20)_ = 0.46, *p* = 0.50), (*F*_(1.20)_ = 0.65, *p* = 0.48), but significant main effects on the CA1.SR volume (*F*_(1.20)_ = 11.51, *p* = 0.003) and (*F*_(1.20)_ = 8.54, *p* = 0.008) were observed. The volume of the hippocampus in the MS, MS.EE, and control.EE groups significantly increased compared to the control group (*p* = 0.01, *p* < 0.001, *p* = 0.002). A significant difference was also found between MS.EE and both MS (*p* = 0.001) and control.EE groups (*p* = 0.01) but no significant change was seen between MS and control.EE (*p* > 0.05) (Figure 5A). The size of CA1.SR area in the MS and MS.EE groups increased significantly in comparison with control group (*p* = 0.04, *p* = 0.001) but there was no significant difference between MS and MS.EE groups (*p* > 0.05) (Figure 5B).

#### 2.4.2. Effects of MS and EE on the Number of Pyramidal Neurons and Volume of the Infra-Limbic and Pre-Limbic Areas

**Figure 6:**
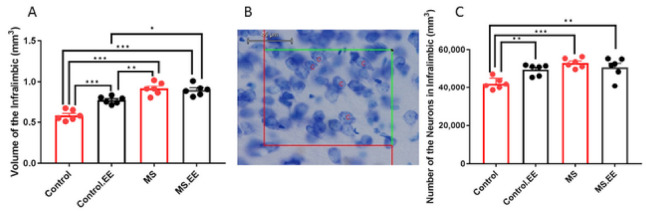
Effects of maternal separation and EE on the (**A**) volume of infralimbic area. (**B**) Illustration of counting the number of neurons in the infra-limbic area with optical fractionator method using objective lens 60× (**C**) Changes in the number of pyramidal neurons in the infra-limbic area of mPFC following MS and EE. * *p* < 0.05, ** *p* < 0.01, *** *p* < 0.001. *N* = 6 in each group. MS: Maternal Separation, EE: Environmental Enrichment.

The result of the volume analysis of the infra-limbic area revealed significant interaction between MS and EE (*F*_(1.20)_ = 14.54, *p* = 0.001) with significant main effects of MS (*F*_(1.20)_ = 71.45, *p* < 0.001) and EE (*F*_(1.20)_ = 9.51, *p* = 0.006). Tukey post hoc analysis showed that the volume of the infra-limbic area in the control.EE, MS, and MS.EE groups was significantly increased in comparison with the control group (*p* < 0.001). There was also a significant difference between MS and control.EE (*p* = 0.006) groups as well as between MS.EE and control.EE groups (*p* = 0.018) (Figure 6A). No significant changes in the volume of the pre-limbic area were detected (*p* > 0.05) (Data were not shown). Regarding changes in the number of pyramidal neurons in the infra-limbic area, there were significant interaction of MS and EE (*F*_(1.20)_ = 10.81, *p* = 0.004) and main effect of MS (*F*_(1.20)_ = 16.48, *p* = 0.001) with no significant main effect of EE (*F*_(1.20)_ = 2.95, *p* = 0.10). Neuronal number in the infra-limbic area significantly increased in control.EE (*p* = 0.01), MS (*p* < 0.001), and MS.EE (*p* = 0.003) groups compared to the control group (Figure 6C). 

### 2.5. Correlations between Behavioral and Biological Parameters 

**Figure 7:**
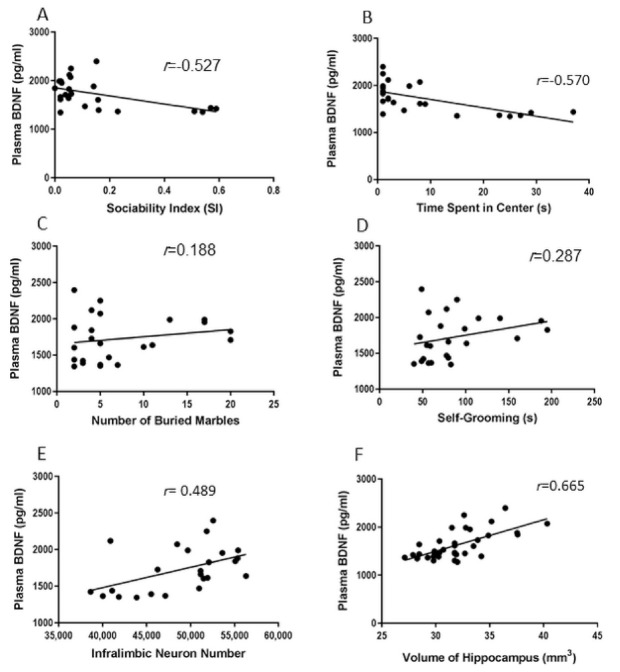
Significant negative correlations between plasma BDNF level and sociability index and time spent in the centre of the open field (*r* = −0.527, *p* = 0.009 and *r* = −0.570, *p* = 0.001) (**A**,**B**). No significant correlation between repetitive behaviours (marble burying and self-grooming) and plasma BDNF level (**C**,**D**) was observed (*p* > 0.05). Significant positive correlations between plasma BDNF level and number of the neurons in infra-limbic and volume of the hippocampus (*r* = 0.489, *p* = 0.015 and *r* = 0.665, *p* < 0.001) (**E**,**F**).

**Figure 8:**
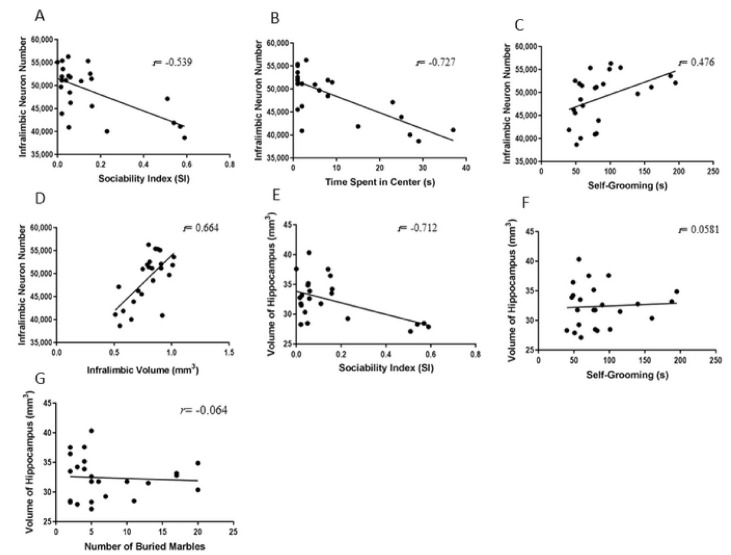
Significant negative correlations between number of the neurons in the infra-limbic area and social index and time spent in centre of the open field (*r* = −0.539, *p* = 0.007 and *r* = −0.727, *p* < 0.001) (**A**,**B**). Significant positive correlation between neuron number in the infra-limbic and self-grooming (*r* = 0.476, *p* = 0.019) (**C**) and infra-limbic volume (*r* = 0.664, *p* < 0.001) (**D**). Significant negative correlation between volume of the hippocampus and sociability index (*r* = −0.712, *p* < 0.001) (**E**). No significant correlation between repetitive behaviours (marble burying and self-grooming) and hippocampus volume (**F**,**G**) were observed (*p* > 0.05).

Two tailed Pearson correlation analysis (Figure 7A,B) showed that sociability index and time spent in the centre of the open field (anxiety behaviour) had significant negative correlations with plasma BDNF level (*r* = −0.527, *p* = 0.009 and *r* = −0.570, *p* = 0.001). Significant negative correlations between number of pyramidal neurons in the infra-limbic area and social index and time spent in centre of the open field (anxiety behaviour) were also detected (*r* = −0.539, *p* = 0.007 and *r* = −0.727, *p* < 0.001) (Figure 8A,B). Significant positive correlations between number of neurons in the infra-limbic, self-grooming (*r* = 0.476, *p* = 0.019), and the volume of the infra-limbic were observed (*r* = 0.664, *p* < 0.001) (Figure 8C,D). There was also significant negative correlation between volume of the hippocampus and sociability index (*r* = −0.712, *p* < 0.001) (Figure 8E). No significant correlations between repetitive behaviours (marble burying and self-grooming) and plasma BDNF level (Figure 7C,D) or hippocampus volume (Figure 8F,G) were observed (*p* > 0.05). Significant positive correlations between plasma BDNF level and number of the neurons in infra-limbic and volume of the hippocampus (*r* = 0.489, *p* = 0.015 and *r* = 0.665, *p* < 0.001) were seen (Figure 7E,F).

### 2.6. Results of the Supplementary Experiment (Treatment with both EE and Oxytocin)

**Figure 9:**
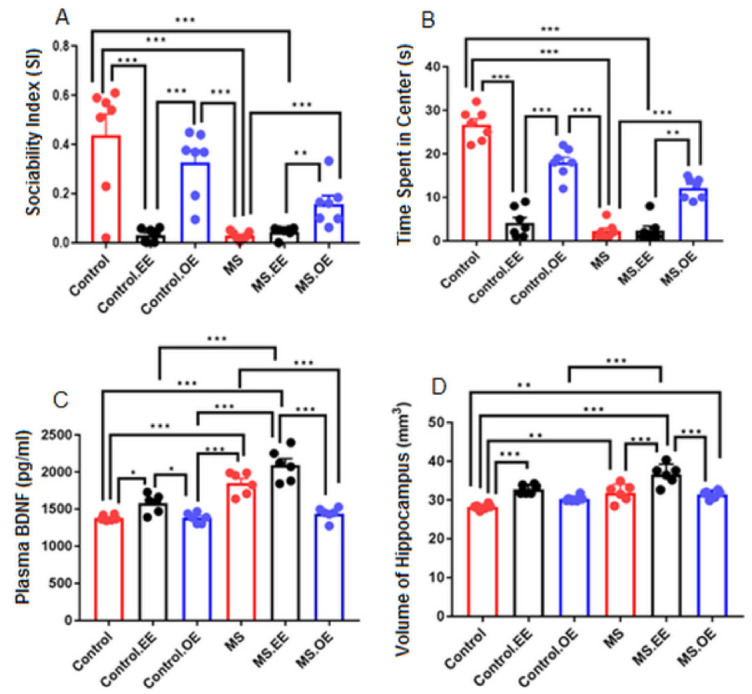
Effects of oxytocin on the negative effects of EE on the social index (*n* = 10) (**A**), anxiety (*n* = 10) (**B**), plasma BDNF level (*n* = 6) (**C**) and volume of the hippocampus (*n* = 6) (**D**). * *p* < 0.05, ** *p* < 0.01, *** *p* < 0.001. Data for plasma BDNF and hippocampus volume are presented before log-transformation, MS: Maternal Separation, EE: Environmental Enrichment.

In this section, we tried to investigate the effect of combination of oxytocin and EE on the autistic-like behaviors in connection with changes in brain plasticity in maternal separation and control groups. As was shown in Figure 9, there was a significant difference between control.OE and both control.EE and MS groups in social index (*p* < 0.001), anxiety behavior (*p* < 0.001), and plasma BDNF level (*p* < 0.05, *p* < 0.001). Moreover, there was a significant difference between MS.OE and both MS.EE and MS in social index *p* < 0.01, (*p* < 0.001) and anxiety behavior (*p* < 0.01, *p* < 0.001). A significant difference between MS.OE and MS.EE was observed in the volume of the hippocampus (*p* < 0.001) and plasma BDNF level (*p* < 0.001). Therefore, our results indicated the possible reversal effect of oxytocin on the adverse effects of EE on the social and anxiety behaviours.

## 3. Discussion

The main finding in the present work was that environmental enrichment, applied after weaning, had dual effects on autism-related behaviors in a rat model of maternal separation. EE reduced repetitive behaviors which were induced by maternal separation, but had no effect on anxiety and social behavioral deficits in the maternal separated group. Similarly, in the control group, environmental enrichment resulted in decreased social behaviors and increased anxiety-like behavior. Quantitative analysis of brain demonstrated that enriched environment increased volume of the hippocampus and the infra-limbic area in parallel with increased number of pyramidal neurons and higher plasma BDNF level. We also demonstrated that oxytocin could ameliorate the adverse effects of EE on anxiety and social behaviors and normalized plasma BDNF levels. 

These findings are in accordance and extend earlier findings, where environmental factors had a key role in the etiology of autism [26,27]. Early life stress, such as low maternal care in rodents, alters the methylation pattern of involved genes in the stress response cascade, and affects hippocampal development which is associated with increased susceptibility to anxiety and depression-like behaviors [28,29]. Studies in rodent offspring found that the mother-pup relationship is crucial for the growth and development of offspring [5]. Indeed, any disruption in this relationship causes disruption in both mother and pup oxytocin systems which finally lead to impaired brain development of the pups [30] and could be one of the mechanisms underlying autism related behavioral following maternal separation. In agreement with our results, the development of autism-like behaviors following adverse early-life experience in rats has been reported in several studies [11,12,31]. 

We detected alternations in plasma BDNF levels and increased neuroplasticity of the hippocampus and infra-limbic brain regions by maternal separation and EE. It is of interest that both MS and EE separately caused an increase in the level of plasma BDNF and the EE effect was further enhanced when combined with MS. Although plasma BDNF level increased in control.EE group compared to the intact control group, there was a significant difference between MS and control.EE groups. On the other hand, there was no significant difference between MS and MS.EE groups. Therefore, MS had a stronger effect on increasing plasma BDNF level than enriching the environment, and this may be one of the reasons for the different behavioral effects of these two factors. Numerous data indicate that BDNF has a critical role in the neuronal plasticity in connection with psychiatric disorders, and changes in the level of neurotrophins following exposure to stressful events in early life, lead to greater sensitivity and vulnerability to mental disorders in adulthood [1,32]. Several studies observed increased levels of BDNF in the plasma of rodents and primates following early life maternal deprivation [33,34]. Positive correlation between peripheral (including plasma and blood) and brain BDNF levels have been observed in rats suggesting that plasma BDNF levels may reflect the BDNF expression in the brain [35]. Therefore, our findings on the neuroplasticity changes could be indirectly due to the BDNF level alternations. In our published study, we found the effect of oxytocin on the improvement of the autistic like behaviors and normalizing the plasma BDNF level in the maternal separated rats (24). In the supplementary study of this work we also found that oxytocin can reverse the elevated plasma BDNF level in the EE groups resulting in the improvement of the behavioral phenotypes. This suggests that BDNF may play an important role in the pathophysiological changes underlying autistic-like behaviors. It is of interest that elevated BDNF levels in the plasma of ASD patients has been reported [36]. Importantly a significant correlation between the severe form of autism and higher level of peripheral BDNF level was reported [37]. It is indicated that BDNF is involved in the survival and differentiation of dopaminergic neurons in the developing brain [38]. Recently, it is also shown that enrichment conditions resulted in decrease in the D2 receptor expression in the hippocampus [39]. Accordingly, modulation of dopaminergic neurons by BDNF could be one of the possible explanations of effect of EE on the autistic like behaviors. It is also shown that the variation, c.Val66Met located in the BDNF gene has been associated with increased BDNF serum concentration in depressed patients [40] and c.Val66Met has identified as a possible genetic modifier of disease severity in Rett syndrome [41].

Recently, it is shown that physical enrichment (PE) versus social enrichment (SE) may differentially influence the synaptic plasticity of brain with the consequence of behavioral alteration. In the environmental enrichment paradigm, the animals are kept in a wide interactive environment with a variety of objects in terms of texture, size and shape that are regularly changed [42,43]. Past studies showed that exposure to an enriched environment in wild type animals improved memory, learning, and reduced anxiety [44,45]. For instance, benefits of EE on the improvement of behavioral impairments such as motor performance, depressive-like behavior, as well as defects in BDNF expression in the hippocampus and striatum in mice models of Huntington’s disease have been effectively shown [46,47]. In the present study, we observed the effect of EE on reducing repetitive behaviors which were induced by maternal separation [48]. The remarkable note in our results was an increase in anxiety and a decrease in the social behavior in rats experiencing the enriched environment. There are conflicting results regarding the effects of environmental enrichment on the autism symptoms. For example, in 2018 a study on the genetic model of Shank3 autism showed the negative effect of environmental enrichment on the anxiety and its lack of impact on social behavior [22]. In 2012 Lacaria et al. stated that although EE was able to show positive effects on some behaviors in mice, it resulted in increased non-contact violence in the direct and inactive social interaction test in normal mice [23]. In a study on the rats, an increase in basal corticosterone concentration following infra-limbic stimulation happened by environmental enrichment [49]. Accordingly, these evidences would help us explaining the increased number of the neurons in the infra-limbic and bigger size of this area along with higher anxiety behavior in EE groups in the present study. However more detailed studies may help to clarify the detailed mechanistic pathway. Although the mechanism of environmental enrichment benefits is not fully understood, it has been suggested that BDNF expression in the hippocampus is enhanced by sustained epigenetic changes of promoters under enriched environment [50] and positive correlation between increased plasma and hippocampus BDNF levels has been seen in rats under the environmental enrichment [51]. Stimulation of BDNF signaling by EE in the hippocampus of mice Alzheimer’s disease model has also been observed by up-regulating BDNF mRNA expression level [52,53]. It is noteworthy that changes in the BDNF level resulted in increased glutamate receptors expression, including AMPA receptor family, glutamate receptor-1 (GluR1), glutamate receptor-2 (GluR2), and NMDA receptors [54,55]. Considering that changes in the glutamate system is implicated in the disruption of the inhibitory excitatory balance of the brain in autism [11], this might be one possible reason for the adverse effects of EE on social behavior. Interestingly, the suppression of the glutamate system in infra-limbic via reducing the release of glutamate by oxytocin has been demonstrated [56], which gives us the possible explanation for the therapeutic effect of oxytocin on the adverse effects of EE.

We believe that following factors about the behavioral results must be considered: 1. The time and duration of the environmental enrichment period. In this study, environmental enrichment started from the day of weaning and continued by the day of behavioral testing (PND42-50). Sudden entry into the enriched environment may act as a trigger for stress. It may be possible to get different results by reducing the enrichment duration that could be considered in the future studies; 2. Over-involvement of animals with objects as a reducing agent of social interaction and that could be an explanation for the decline in social behavior in the EE groups; 3. Excessive stimulation by changing the objects every five days during EE might cause psychological overloading and in turn behavioral abnormalities; 4. Increasing in the number of animals, even with larger cages, may increase competition by exacerbating violent behaviors associated with increased anxiety and a decreased tendency for social behavior. Despite the general categorization of special environmental conditions as harmful and/or beneficial to health, one of the challenges in psychiatric research is that not all individuals exhibit the same reactions [57]. Therefore, it requires further investigations to find the mechanism of action and define a standard method with precise determination of its various features to the correct and beneficial application. 

The complex and rather unknown pathophysiology of ASD is one of the limitations of the related research particularly in animal models. Difference in the environmental enrichment in laboratories such as size of the cage, number of the animals, playing equipment, frequency of object changing are other limitations of the current work which make the direct comparisons and conclusion complex.

## 4. Material and Methods 

### 4.1. Animals and Maternal Separation

Animals were kept at 21 °C, humidity (50%) and 12/12 light-dark cycle (lights on at 7:00 a.m.) with free access to food (Pellets, Pars Animal Feed Co., Iran) and water during the experiment. Male and female wistar rats (260–290 g and 10–11-week old, *n* = 10 in each group) were purchased from School of Biology, Shahid Beheshti University (Iran), and used for mating. The animals were housed in cages (26.5 × 42 × 15 cm^3^ in size and *n* = 4–5 per cage) and allowed to acclimatize in the animal colony for two weeks before any procedures commenced. Male and female rats were randomly paired during 5 days in the cages in the size of 26.5 × 42 × 15 cm^3^ allowing them to mate. Nine pregnant rats were kept individually in the same size of cages for one week before giving birth (Gestational day (GD) 14–21). Subsequently, male pups (*n* = 40) from 75 litters were randomly divided into two groups: maternal separation (MS) and control. In the maternal separation group, pups were separated from their mother for 3 h (9–12 a.m.) at PND (post-natal day) 1–14, placed individually in small cages (24 × 13.5 × 13 cm^3^; about 4 m far from the mother). From PND15–21, the rats remained with their mothers. The pups in the control group were normally raised with the mother until weaning age. At PND21, the rats of both groups were equally divided into two subgroups of either environmental enrichment (EE) or normal conditions. Accordingly, the study consisted of 4 groups (10 rats in each group) as follows: Control; control.EE; MS; MS.EE. 

This research was conducted in accordance with Ethics Committee standards of Shahid Beheshti University (IR.SBU.ICBS.97.1045). 

### 4.2. Environmental Enrichment 

**Figure 10:**
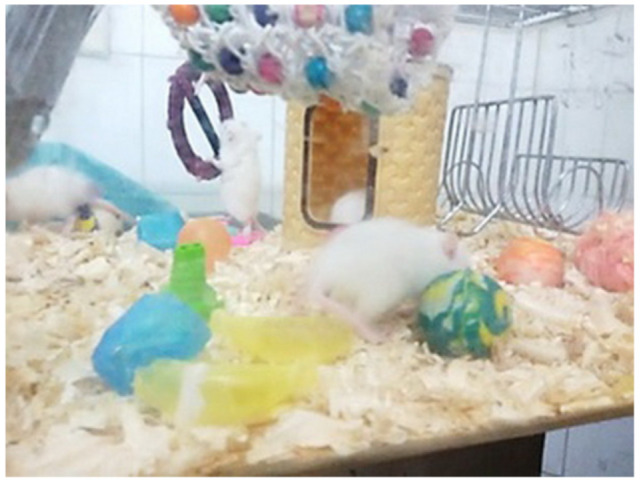
Example of environmental enrichment: after weaning at PND21, rats were kept in large cages with a greater number of rats, different objects and playing equipment which were changed every 5 days, until the behavioral test day.

Two groups of animals were randomly selected among the pups (control.EE and MS.EE) and underwent environmental enrichment after weaning (PND21). The rats were housed in large cages (50 × 80 × 30 cm^3^) with a greater number of rats (*n* = 8–9) in each cage, and provided with different objects and playing equipment (Figure 10). These objects were changed every 5 days until the behavioral test day. The other two groups without treatment (control and MS) were kept under the normal housing conditions (cages with 26.5 × 42 × 15 cm^3^ in size and *n* = 4 in each cage) with no enrichment of environment. 

### 4.3. Supplementary Experiment

After completing the experiments and considering the results given below, as well as the results of previous work in which we showed the effect of oxytocin in improving autism-related symptoms, we decided to investigate the combined effect of oxytocin and environmental enrichment (OE) on some parameters which were altered unexpectedly following EE. For this purpose, two other groups of rats (control.OE and MS.OE) were defined, which received both oxytocin and EE treatments. Oxytocin (O3251Sigma) was administered intraperitoneally (ip) in a dose of 1 mg/kg from PND22 to PND30 (with 48 h interval) and EE was applied as described above.

### 4.4. Behavioral Tests

At adolescence (PND42-50), sociability, repetitive, anxiety-like behaviors, and motor activity were assessed using the three-chamber, marble burying, and open-field tests, respectively. The rats were transferred to the behavioral test room one hour before starting the tests, and all tests were performed in a dimly lit room (20 lux) during one day (8 a.m. 3 p.m.) in the following order to decrease the amount of stress: (1) Three-chamber test; (2) Open field test, and (3) Marble burying test. The test boxes were cleaned with 70% alcohol between each test. Each day, four rats were tested, with all tests recorded with cameras on top of the boxes (80 cm above the floor). Details of the behavioral tests have been described previously [24]. 

#### 4.4.1. Three-Chamber Test 

Briefly, the three-chamber test is used for examining the social behavior in rodents [58]. The test rat was placed for 5 min in the middle chamber of a three-chamber box (20 × 35 × 40 cm^3^ for each chamber) with the chambers connected to each other with glass walls. Thereafter, the rat freely moved and habituated to the all chambers for 10 min. Subsequently, a stranger rat of the same age and sex with no previous contact with the test rat was placed in one of the side chambers, and in the other side of the chamber, an empty cage was placed as a novel object. Finally, the doors were simultaneously opened, and the test rat had 10 min to freely move between the three chambers. At the end of the test, the rat was transferred to the resting cage and was given an hour before going through the next test. The following parameters were recorded in the three-chamber test: time spent in the stranger rat chamber, time spent in the novel object chamber, time spent sniffing stranger rat, and time spent sniffing novel object. The criterion for sniffing time was the nose directly toward the stranger rat or empty cage.

#### 4.4.2. Open Field Test

In the open-field test, the rat was placed for 15 min in a box (40 × 40 × 40 cm^3^) which was divided by lines into the central circle and squares around it [11]. The following parameters were recorded in this test: time spent in the central circle, time spent for self-grooming, number of times the rat crossed the lines. The rats were then returned to the resting cage and marble burying test was performed after an hour of resting.

#### 4.4.3. Marble Burying Test

This test was performed for 30 min in a (40 × 40 × 40 cm^3^) box with 20 glass marbles arranged in 5 rows. After 30 min of test, the number of hidden marbles (75% covered with bedding) was considered as the stereotype behavior [59].

#### 4.4.4. Tissue Preparation

Thirty minutes after completing behavioral tests, the rats were deeply anesthetized with ketamine (Ratiopharm, Germany; 150 mg/kg, i.p) and blood samples were taken directly from heart using EDTA-covered tubes. The blood samples were centrifuged at 6000 rpm for 20 min and the supernatants were stored at −80 °C until further processing. Subsequently, transcardial perfusion was performed with ice cold saline followed by 4% paraformaldehyde in 0.1 M phosphate buffer (PB, pH 7.4) for 10 min. The brains were then extracted and stored in the same fixative solution at 4 °C until further processing. At the time of tissue analysis, right or left hemispheres of the brains were randomly selected and kept in 30% sucrose for 72 h, then frozen in liquid nitrogen and coronally cut at 50-µm thickness on cryostat cutting devise (SCILab, Cool-Cut, SCI85683, England). Using a systematic sampling principle, the systematic sampling fraction (SSF) for hippocampus and medial prefrontal cortex were 1/12 and 1/3, respectively. The first section of each region was selected using a random number table. One series of brain sections was Nissl stained with 0.25% thionin (Sigma T3387).

#### 4.4.5. Plasma BDNF Level Assay 

Plasma BDNF level was assessed according to the manufacturer’s description. Briefly, using ELISA kits (Sigma RAB 1138), the plasma samples were diluted and applied in 1:300 with the standard curve in the range of 12–3000 pg./mL. The reaction rate was measured at 450 nm using a microplate reader (Biotech, Oklahoma City, OK, USA, 100 Tigan St, Winooski, VT 05404, USA). 

### 4.5. Three-D Quantitative Measurements of the Brain

#### Volume Estimation 

Volume of the hippocampus and its sub-regions including granular cell layer (GCl), molecular layer of dentate gyrus (MDG) and CA1 stratum radiatum (CA1.SR), and infra-limbic and pre-limbic areas of the mPFC were measured using the Cavalieri estimator with point counting, and the newCAST software (Visiopharm, Hørsholm, Denmark) and light microscope (Olympus, BX56) modified for stereology with motorized stage (H1P4BX ProScan stage with V31XYZE ProScan III Controller). Delineation of area of interest was performed using an objective lens 4× and the volume measurement was done using an objective lens 10×. To calculate the volume of brain regions following formula was used [60,61]:

$$V=\sum P\;\cdot \;\left(\frac{a}{p}\right)\;\cdot \;T\;\cdot \;\frac{1}{SSF}$$

where ∑P was the total number of the points counted in the delineated regions per animal, (*a/p*) was the area per test point; *T* is the section thickness (50-µm), and *SSF* was the section sampling fraction (1/12 for hippocampus and subregions and 1/3 for mPFC subregions). 

### 4.6. Estimation of the Pyramidal Neuron Number

The optical fractionator method was applied by using 60× oil-immersed objective lens in order to count the number of pyramidal neurons in the infralimbic area. The effect of section shrinkage during tissue preparation was corrected based on the optimal disector height determination by counting the number of neurons through the full thickness of nine brain sections from the infralimbic area (with systematic random sampling the sections between animals). In the thickness from 5-µm to 15-µm (10-µm thickness) a constant cell density was seen using Z-plot (histogram) of the counted neurons. The criterion for counting the pyramidal neurons was the nucleolus being in focus and fully or partially inside the unbiased counting frame without touching the forbidden lines. The following formula was used to calculate the total number of pyramidal neurons:

$$N=\frac{1}{SSF}\;\cdot \;\frac{1}{ASF}\;\cdot \;\frac{1}{HSF}\;\cdot \;\sum Q-$$

where *N* was the total number of pyramidal neurons in infralimbic area; *∑Q−* was the number of the counted pyramidal neurons; *SSF* was the section sampling fraction (1/3); *ASF* was an area sampling fraction; and *HSF* was the height sampling fraction (mean of the *Q−* weighted height of the dissector) [62].

### 4.7. Statistical Analysis 

Statistical analysis was performed using SPSS software (IBM SPSS Statistics for Windows, Version 24.0. Armonk, NY: IBM Corp) and plots were generated using GraphPad Prism version 8 (GraphPad software, San Diego, CA, USA). A Q-Q plot was generated to check the normality of data distribution. Equality of variances was assessed by Leven’s test. In case of inequality of variances, logarithmic transformation was performed on the original data. Within group comparison was performed for social behaviors by repeated-measures analysis of variance (ANOVA) [24]. Other data were analyzed using two-way ANOVA followed by Tukey post-hoc test. Two tailed Pearson analysis was used to find the correlation between variables. Data were presented as mean ± SEM. The null hypothesis was rejected with *p* < 0.05 as the level of significance. 

## 5. Conclusions

The current study indicates that environmental enrichment ameliorates repetitive behaviors in a maternal separation rat model of autism but caused increased anxiety and social behaviors deficits alongside with elevated plasma BDNF levels which was associated with plasticity changes in the hippocampus and infra-limbic cortical area. BDNF can be highlighted as a mediator of effects of EE on social and anxiety behaviors as well as brain plasticity changes. It seems that changes in the plasma BDNF level had no relation with repetitive behaviors indicating the different involved mechanism in repetitive behavior than the mechanisms underlying social behavioral deficits in ASD which requires further studies to achieve certainty. Taken together, our results suggest that therapeutic approach of environmental enrichment still needs more investigations to find the answer to the open questions on some negative effects.

## Data Availability

The data presented in this study are available on request from the corresponding author. The data are not publicly available due tosome further analysis might be done to publish another paper from the data.
